# Adherence Support Workers: A Way to Address Human Resource Constraints in Antiretroviral Treatment Programs in the Public Health Setting in Zambia

**DOI:** 10.1371/journal.pone.0002204

**Published:** 2008-05-21

**Authors:** Kwasi E. Torpey, Mushota E. Kabaso, Liya N. Mutale, Mpuma K. Kamanga, Albert J. Mwango, James Simpungwe, Chiho Suzuki, Ya Diul Mukadi

**Affiliations:** 1 Family Health International/Zambia Prevention Care and Treatment Partnership, Lusaka, Zambia; 2 Ministry of Health, Lusaka, Zambia; 3 Family Health International, Arlington, Virginia, United States of America; McGill University, Canada

## Abstract

**Background:**

In order to address staff shortages and improve adherence counseling for people on antiretroviral therapy (ART), the Zambia Prevention, Care and Treatment Partnership (ZPCT) developed an innovative strategy of training community volunteers to provide adherence support at the health facility and community levels. The objective of this study was to assess the effectiveness of these ‘adherence support workers’ (ASWs) in adherence counseling, treatment retention and addressing inadequate human resources at health facilities.

**Methodology/Principal Findings:**

The study used quantitative and qualitative research techniques at five selected ART sites in four provinces in Zambia. Five hundred patients on ART were interviewed using a structured questionnaire to compare the quality of adherence counseling before and after the ASW scheme was introduced at the selected sites and between ASWs and HCWs after the introduction of ASWs. In addition, 3,903 and 4,972 electronic records of all new patients accessing antiretroviral therapy for the time period of 12 months before and 12 months after the introduction of ASWs respectively, were analyzed to assess loss to follow-up rates. Two focus group discussions with ASWs and health care workers (HCWs) were conducted in each clinic. Key informant interviews in the ART clinics were also conducted. There was a marked shift of workload from HCWs to ASWs without any compromise in the quality of counseling. Quality of adherence counseling by ASWs was comparable to HCWs after their introduction. The findings suggest that the deployment of ASWs helped reduce waiting times for adherence counseling. Loss to follow-up rates of new clients declined from 15% to 0% after the deployment of ASWs.

**Conclusion:**

Adherence counseling tasks can be shifted to lay cadres like ASWs without compromising the quality of counseling. Follow-up of clients by ASWs within the community is necessary to improve retention of clients on ART.

## Introduction

Zambia is amongst the countries hardest hit by the HIV/AIDS epidemic in Africa. It is estimated that 1.2 million of the total Zambian population of ten million was infected with HIV by 2005 [1], [2]. Although declining HIV trends have been observed in young people since 1998, HIV/AIDS in Zambia is still a major threat to the lives of adults of reproductive age and their children [3]. Increasing access to highly active antiretroviral therapy (HAART) could alter this trend [4]–[6].

The efficacy of combination antiretroviral drugs (ARVs) for the treatment of HIV-disease is now well documented [4], [7]–[10]. HAART, involving the use of several medications at a time, has become the standard practice to achieve viral suppression [4], [7]–[9], [11]–[13]. Adherence is closely associated with improved viral suppression, prevention of resistance, delay in disease progression and decreased mortality [7], [8], [11], [14]–[17]. The public health consequence of treatment failure is the possible spread of drug resistant virus strains in the community [9], [18]–[20]. In addition, there are serious public health cost implications as patients are shifted from less costly first line to expensive second line regimens [13].

Shortages of health care workers (HCWs) have been a bottleneck in ART roll out in resource-limited settings. The World Health Organization/Ministry of Health establishment recommends a staff population ratio of 1: 5000 , 1:700, 1: 8000 for doctors , nurses and pharmacists respectively. The existing, human resource capacity in Zambia is far below the recommended cadre to population ratios with existing levels of 1:17,589, 1: 8,064 and 1:473,450 for doctors, nurses and pharmacists respectively [21]. With the rapid expansion of access to ART, the increasing patient load will put a strain in the existing fragile human resource base.

In July 2006, Family Health International's Zambia Prevention Care and Treatment Partnership (ZPCT), funded by the United States Agency for International Development (USAID) through the U.S. President's Emergency Plan for AIDS Relief, developed a ten-day training curriculum to train community volunteers as adherence support workers (ASWs) in order to complement the efforts of the HCWs and help reduce their workload [22]. The ten-day training includes two days of practicum. Participants are observed and assessed by HCWs who are trainers or trained in HIV/AIDS care and ART management and they give feedback to the training facilitators on their performance for purposes of certification. The curriculum is a longer version of adherence counseling training for HCWs but simplified for lay providers. It was implemented after an initial pilot study in two provinces. Apart from technical information on ART and adherence, additional modules on techniques for relationship building, counseling skills and documentation were included. The training modules involve didactic sessions, role plays, group and individual exercises.

Adherence counseling was mostly done by HCWs prior to the introduction of ASWs with other untrained staff like registration officers and data entry clerks helping out in situations of staff shortages. Since July 2006, ZPCT has deployed ASWs to provide adherence counseling to patients, in conjunction with HCWs, as part of the ART team in health facilities. They work alongside nurses and doctors in the ART clinic and are supervised by the ART Adherence Counselor who is a Health Care Worker (HCW) and are expected to participate in consultations and meetings as part of the ART Team. ASWs are mostly persons living with HIV/AIDS (PLWHA), often on ART themselves. ASWs also conduct community visits to track down patients who have missed their clinic appointments and provide support to improve adherence.

ART programs using community workers and treatment supporters to implement HAART using the Directly Observed Therapy (DOT) approach within the community have reported some success [12], [23]–[28]. While the predictors and biologic consequences of non-adherence to ART are well documented, there is a general lack of information comparing the quality of adherence counseling provided by lay providers such as ASWs with HCWs [23], [29], [30]. There is also a paucity of information on the impact of using lay providers such as ASWs in both the health facility and community settings, even though these can be vital strategies in ART program implementation. In this study, we aimed to understand the effect of ASWs in addressing the human resource gaps and loss to follow up rates. Furthermore, we assessed the quality of adherence counseling before and after the introduction of ASWs and also compared quality of adherence counseling by ASWs and HCWS post ASW intervention. This study, the first of its kind in Zambia, contributes to understanding how effective lay providers such as ASWs are in providing quality adherence counseling services, reducing loss to follow rates and HCWs' workload, not only in the community but as part of the ART clinical team in the health facilities.

## Methods

### Study design

The study was performed from 1^st^ March to 4^th^ April 2007. The quantitative component combined a cross-sectional study with retrospective patient records review supplemented by qualitative data obtained from focus group discussions (FGD) and key informant interviews at the five selected ART sites in Zambia. Both qualitative and quantitative components of the study were conducted during the study period.

### Quantitative data collection: Cross sectional study

The cross sectional study involved interviewing 500 ART clients accessing services at five public health facilities. Information was collected through a structured questionnaire, which included questions pertaining to patients' social demographic characteristics, period when the patients started ART, whether adherence counseling was offered and by whom. Quality of adherence counseling such as (1) duration of the counseling sessions, (2) counseling procedures, (3) counselors' responsiveness to clients' questions, (4) coverage of key questions and information including the advantages of ART, drug-specific information, and safe sex practices were measured as well as the patients' adherence to drugs (based on patients' self-report) were explored. ART patients who enrolled in the ART program before July 1, 2006 were categorized as “before ASW intervention”. Those that enrolled after July 1, 2006 were classified as “after ASW intervention”. The questionnaire did not collect information on disease severity, such as clinical staging, CD4 count and viral load of the ART clients.

#### Sample size determination

The minimum sample size of ART patients of 484 was determined with the aim of detecting 26% differences in the counseling and adherence outcomes between those patients who were counseled by HCWs before the introduction of ASWs and those who were counseled both by ASWs and HCWs after ASWs were deployed. Several outcomes were set at 50% as there were no reliable estimates of the counseling and adherence outcomes at the time of the study. The level of significance was set at 5%, and the power was set at 80%. Table 1 outlines options of variables occurring at different frequencies, differences detected and estimated minimum sample size.

**Table 1:** Differences in proportions that can be detected in studies with varied sample sizes for variables occurring at various baseline frequencies

Alpha (α)	Power (1-β)	Frequency of variable (P)	Difference that can be detected	Precise sample size required to detect this difference
0.05	0.80	10%	90%	522
0.05	0.80	25%	48%	496
***0.05***	***0.80***	***50%***	***26%***	***484***
0.05	0.80	75%	13%	540
0.05	0.80	90%	8%	442

#### Eligibility criteria

Criteria for eligibility for patients on ART were those who were aged 18 years or older and had commenced ART from January 2006 onwards. Eligible patients on ART were approached consecutively during the days the ART clinics were in operation. The patients were recruited to participate in the study as they were exiting from the ART clinic until the last patient was seen by the health care worker at the site. This process occurred over several clinic days and up to four weeks until the required sample size was reached.

### Quantitative data collection: Retrospective review of patient records

3,903 and 4,972 electronic patient records of all new patients on ART from the ARV dispensing tool were analyzed for the time period of 12 months before and 12 months after the introduction of ASWs to determine the ARV treatment retention rates. The ARV dispensing tool (developed by Management Sciences for Health's Rationale Pharmaceutical Management Plus Project funded by USAID) is a Microsoft Access electronic database used to track dispensing of ARVs, appointments, loss to follow-up and treatment retention rates. Loss to follow-up was defined as missing three consecutive pharmacy appointments.

### Qualitative data collection

At each study site, two FGDs were held, one with HCWs and the other with ASWs. HCWs who were providing ART services at least twice a week and had been working at the site for at least 12 months were eligible to participate. All ASWs at the study sites were eligible to take part in the FGDs. At each of the study sites eligible participants were identified and then given appointments to attend the focus group sessions. Each of the focus group discussions was facilitated by a trained research assistant and a co-moderator who took detailed field notes of the discussions. Printed reports were prepared from the field notes immediately after the discussions. This data was collected during the same time period as the quantitative data. Themes explored during the FGD centered on strengths and weaknesses of the ASW scheme, and recommendations on issues such as the role ASWs in addressing the human resource shortage problem, quality of adherence counseling and patient waiting times.

Key informant interviews using a set of questions to guide the discussions were conducted with the facility manager or in-charge of the ART clinic to gain their perspective on the ASW scheme. The questions focused mainly on the effect ASWs had in relieving HCWs time to attend to other activities, waiting times experienced by patients and quality of counseling in general.

### Study setting and site selection

The study was conducted at five ART sites in four ZPCT-supported provinces as follows: Kasama General Hospital in Northern Province, Mansa General Hospital in Luapula Province, Kabwe Mine Hospital in Central Province, and Ndola and Kitwe Central Hospitals in Copperbelt Province.

These five sites were purposively selected from the 49 ZPCT supported sites offering ART services during that time period. The selection was based on the availability of the electronic ARV dispensing tool which captured data at least 12 months before and 12 months after the deployment of the ASWs.

### Data analysis

The data was analyzed using STATA Version 9.2 (STATA Corp, TX, U.S.A) to compare proportions between before and after the introduction of ASWs as well as between HCWs and ASWs after introduction of ASWs. Z-score was calculated to compare differences between groups, and whether the difference was statistically significant (p<0.05). This has an advantage over the chi-squared test (test of association), because z-test enables one to determine whether the observed differences or changes (e.g. increase or reduction) are statistically significant. Therefore we were able to compare outcomes before and after the ASW scheme and between ASWs and HCWs post ASW intervention.

Data captured from the computerized ARV dispensing tool was analyzed to determine the proportion of new patients who were lost to follow-up and those who remained on ART before and after the introduction of ASWs.

The narratives from the FGDs with HCWs and ASWs were analyzed through an inductive approach. Field notes from the FGDs were electronically transcribed to carry out an in-depth analysis of the narratives obtained from the FGDs. The transcripts were then reviewed for accuracy and completeness and then coded according to key themes related to the study objectives. Selected quotations were identified to illustrate the common themes that arose from the series of FGDs and presented in this paper.

### Protection of Human Subjects and Ethical approval

The study was explained to the ART clients and oral informed consent was obtained to address issues of identification and perceptions and implications for confidentiality, stigma and discrimination. A similar process was followed in the focus group discussions and oral informed consent obtained from both ASWs and HCWs. Participants were enrolled only after consent was provided. Written informed consent was obtained from facility managers/ART clinic in-charge who participated in the key informant interviews.

Approval to conduct the study was granted by the Zambia Research Ethics Committee of the University of Zambia and the Protection of Human Subject Committee of Family Health International in North Carolina, U.S.A.

## Results

### Analysis of quantitative data

#### Socio-demographic characteristics of ART patients interviewed

The 500 clients on ART interviewed included 295 females (59.0%) and 205 males (41.0%). Most of the patients were in the age group of 35–49 years (42.8%) followed by 25–34 years (38.8%). The mean and median age was 36 and 35 years respectively. Almost half of respondents (48.0%) were married and in a monogamous relationship while 2.0% were in polygamous relationships. 35.0% were divorced, separated or widowed.

47.4% of the ART patients interviewed attended school through the secondary level, 30.2% received only primary education, and 3.6% had no formal education. 276 clients (55.2%) started ART before the introduction of the ASW program, while 224 (44.8%) started ART after the inception of the program.

#### Pre-treatment and follow-up adherence counseling

**Figure 1:**
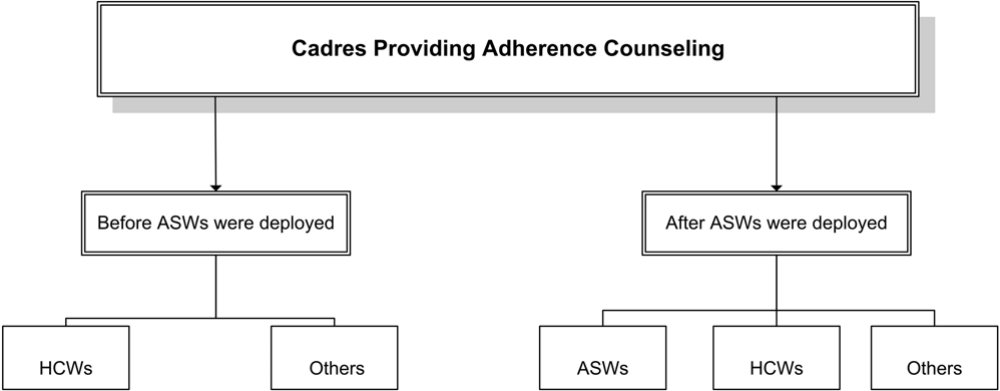
Type of Cadres Providing Adherence Counseling Services. This figure shows the type of cadres that were providing adherence counseling services before and after adherence support workers (ASWs) were deployed in the health facilities. Health Care Workers (HCWs) and other untrained staff such as data entry clerks, registration officers provided adherence counseling prior to the ASW intervention. After ASWs were introduced, they joined the above mentioned cadres in providing adherence counseling services.

Figure 1 outlines the various cadres that provided adherence counseling services before and after ASWs were deployed into the facilities.

Table 2 indicates the proportion of enrolled ART participants that had received pre-treatment and follow-up adherence counseling before and after the ASW program was introduced.

**Table 2:** Proportion of ART clients receiving pre-treatment and follow-up adherence counseling before and after ASW were introduced

	Before ASWs (N = 276)	After ASWs (N = 224)	P value	95% Confidence Interval
Percentage of ART clients who received pre-treatment adherence counseling	89.1.%	90.2%	>0.05	−0.64; 0.04
Percentage of ART client who received follow-up adherence counseling	72.8%	73.7%	>0.05	−0.09;0.07

The data indicates that 89.1% of patients received pre-treatment adherence counseling before the ASW program was initiated, compared to 90.2% after the program was initiated. This difference is not statistically significant (p>0.05). The pattern was similar for follow-up adherence counseling.

There was a significant shift in the cadres providing adherence counseling after the introduction of ASWs. Table 3, shows that the responsibility for pre-treatment adherence counseling shifted from HCWs to ASWs; 72.4% of ART clients received counseling by HCWs before the ASW program, which declined to 52.0%. In table 4, a similar pattern was observed with follow-up adherence counseling, with a decline of 63.0% to 42.4% in the percentage of patients receiving follow-up counseling by HCWs. Similar results were seen when the analysis was disaggregated by ART site and these results were statistically significant (p<0.05). The number of untrained non-health care workers (others) who conducted adherence counseling to support HCWs declined from 37.0% to 2.4% after the introduction of ASWs (p<0.05).

**Table 3:** Cadres providing pre-treatment adherence counseling before and after ASWs were introduced

Provider (Pre-Treatment)	Before ASWs (N = 246)	After ASWs (N = 202)	P value	95% Confidence Interval
HCWs	72.4%	52.0%	<0.05	0.12;0.29
ASWs	0.0%	45.0%	<0.05	−0.52;−0.38
Others*	27.6%	3.0%	<0.05	0.15;0.29
Total	100.0%	100.0%		

* Others–Include data clerks, registration officers etc.

**Table 4:** Cadres providing follow-up counseling before and after ASWs were introduced

Provider (Follow-up)	Before ASWs (N = 200)	After ASWs (N = 165)	P value	95% Confidence Interval
HCWs	63.0%	42.4%	<0.05	0.10;0.31
ASWs	0.0%	55.2%	<0.05	−0.63;−0.48
Others	37.0%	2.4%	<0.05	0.27;0.42
Total	100.0%	100.0%		

#### Quality of adherence counseling and adherence measurement using self-report

**Figure 2:**
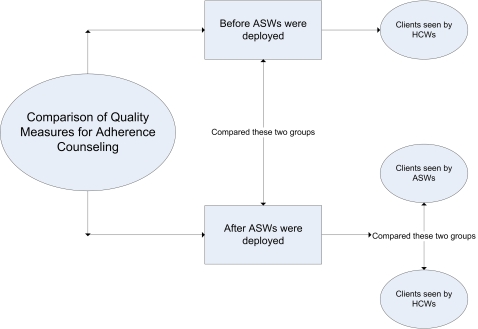
Figure depicting the groups compared for Quality Measures. Various quality measures for HCWs and ASWs were compared for clients who were counseled before and after ASWs were introduced in the facilities. Another comparison was conducted to compare ASWs and HCWs after the introduction of ASWs.

As illustrated in figure 2, comparisons of various quality measures and adherence were done at two levels. Outcomes of ART patients who initiated therapy before the ASW scheme were compared to those who started treatment after the program. Secondly ART patients counseled by ASWs were compared to those counseled by HCW after the introduction of ASWs in the facilities.

#### Duration of adherence counseling

The respondents scored the duration of the counseling as too short, too long or about the right amount of time.

As shown in Table 5, there was an overall increase in the percentage of patients who thought that the time spent on adherence counseling was just right. 77.1% of ART patients thought that the duration of counseling provided by HCWs was just right prior to the intervention, which increased to 81.1% after the intervention. 82.1% of ART patients felt that the duration of counseling provided by ASWs was just right. There is no statistically significant difference in the perception of counseling duration provided by ASWs versus HCWs (p>0.05).

**Table 5:** Results of adherence quality proxy measures before and after ASWs

Quality Measures	Before ASWs	After ASWs	P-value	95% Confidence Interval
Right amount of time spent on adherence counseling	77.1% (N = 201)	81.1% (N = 164)	>0.05	−0.12;0.04
Allowed adherence counseling questions	94.7% (N = 133)	97.3% (N = 112)	>0.05	−0.07;0.02
Provider responded to adherence counseling questions	94.0% (N = 133)	97.3% (N = 110)	>0.05	−0.08;0.02
Easy to understand adherence counseling	95.0% (N = 201)	96.4% (N = 165)	>0.05	−0.05;0.03
Able to mention 2 advantages or more	90.9% (N = 276)	94.6% (N = 224)	>0.05	−0.08;0.01
Able to mention 2 or more drug specific information	85.5% (N = 276)	85.7% (N = 224)	>0.05	−0.06;0.06
Able to mention 2 or more methods of safe sex	86.2% (N = 276)	85.3% (N = 224)	>0.05	−0.05;0.07
Take medicines regularly each day without missing any pill	86.1% (N = 276)	82.1% (N = 224)	>0.05	−0.02;0.10

#### Questions, responses and understanding adherence counseling

The responses indicate that there was no difference in the counseling received before and after the introduction of ASWs (p>0.05)

#### Advantages of ART, drug specific information and safer sex methods

There was no difference in the accuracy of the responses provided before and after the introduction of ASWs across all the elements.

#### Comparing adherence counseling by health care workers and ASWs

As indicated in Table 6, data was disaggregated for ART patients counseled by HCWs after the ASW intervention and compared to those counseled by ASWs across the quality elements above to determine differences. Quality of adherence counseling provided by ASWs was similar to HCW across all the elements, except responding to questions, particularly on advantages of ART, where the ASWs fared better.

**Table 6:** Results of adherence quality proxy measures between HCWs and ASWs after ASWs introduction

Quality Measures	After introduction of ASWs		P-value	95% Confidence Interval
	*HCWs*	*ASWs*		
Right amount of time spent on follow-up adherence counseling	80.0% (N = 70)	82.2 % (N = 90)	>0.05	−0.14;0.10
Allowed adherence counseling questions	97.8% (N = 46)	96.9% (N = 64)	>0.05	−0.05;0.07
Provider responded to adherence counseling questions	*93.5% (N = 46)*	*100.0% (N = 62)*	*<0.05*	*−0.14;0.01*
Easy to understand adherence counseling	94.3% (N = 70)	98.9% (N = 90)	>0.05	−0.10;0.12
Able to mention 2 advantages of ART or more	*88.6% (N = 70)*	*96.7% (N = 91)*	*<0.05*	*−0.16;0.00*
Able to mention 2 or more drug specific information	88.6% (N = 70)	90.1% (N = 91)	>0.05	−0.11;0.08
Able to mention 2 or more methods of safe sex	82.9% (N = 70)	87.9% (N = 91)	>0.05	−0.16;0.06
Take medicines regularly each day without missing any pills	87.1% (N = 70)	86.8% (N = 91)	>0.05	−0.10;0.11

#### Adherence measurement using self-report

Using a three-day recall, ART patients were asked if they took their medicines regularly without missing any pills during that period. There was no statistically significant difference in self-reported adherence before and after the introduction of ASWs (p>0.05). Self-reported adherence between clients counseled by HCWs compared to ASW after the ASW intervention was similar and not statistically significant (p>0.05).

#### Retention and loss to follow-up rates

Data from the ARV dispensing tools was analyzed to determine loss to follow-up and retention rates for all new patients who began ART 12 months before and after the introduction of ASWs. 3,903 electronic records of all new ART patients who commenced treatment 12 months prior to the introduction of ASWs were reviewed. 3,332 patients (85.4%) were retained in the ART program and 571 patients (14.7%) were lost to follow-up over a 12-month period.

4,972 electronic records of all new ART patients who commenced treatment after the introduction of ASWs were also reviewed. 4,972 (100.0%) of the patients had been retained in the ART program in 12 months. This highlights the ability of ASWs to follow up within the community.

### Analysis of qualitative data

The qualitative analysis involved separate FGDs with HCWs and ASWs as well as key informant interviews with facility managers or ART clinic in-charges.

#### Addressing the human resource problem

The FGDs regarding the use of ASWs revealed the following recurring thematic areas related to human resources:

- reduced waiting times;
- reduced workload for HCWs; and
- streamlining patient flow.

#### Addressing patient waiting times

Both the HCWs and ASWs FGDs emphasized the positive role that ASWs are playing in addressing the human resource shortages at health facilities. The overwhelming response from both FGDs was that ASWs have greatly helped to reduce waiting times for patients. The following comments reflect *these perceptions:*

- *“With the placement of ASWs in ART sites, ART clients' waiting time of pre-ART and post-ART adherence counseling has been drastically reduced.” (HCW FGD)*
- *“The presence of ASWs has led to decline of long lines of patients waiting to be reviewed.” (ASW FGD)*
- *“ASWs have shortened the doctors/HCWs time in explaining to clients, leading to reduced waiting times and hence they can see more patients” (HCW FGD)*
- *“Because ASWs spend more time with clients, clinicians can now reduce waiting times for patients.” (HCW FGD)*
- *“Patient waiting time is reduced for there are always two or three ASWs present to provide adherence counseling to ART clients. They have to wait for less than 10 minutes to be served at the pharmacies.” (ASW FGD)*

#### Reduced workload for HCWs

The shifting of tasks from HCWs to ASWs has not only relieved HCWs but has also allowed them to be reassigned to other duties. The following quotes from both HCWs and ASWs FGD put it succinctly:

- *“HCWs/nurses who used to be involved in adherence counseling have been relieved to attend to other duties. Human resource has been freed and has been allocated elsewhere.” (HCW FGD)*
- *“Some clients tend to be more open about their health problems to ASWs as they have the same HIV status.” (ASW FGD)*

#### Streamlining patient flow

In addition, ASWs have helped to streamline the patient flow by quickly identifying patients, leading to a greater number of clients being seen.

- *“ASWs know the patient flow system and documentation process and therefore assist patients to be attended to quickly.” (ASW FGD)*
- *“ASWs are able to identify new and old clients and thus are able to deal with new clients more promptly.” (HCW FGD)*
- *“More clients are being seen by clinicians since waiting time has reduced.” (HCW FGD)*

#### Addressing the quality of adherence counseling

The analysis of qualitative data shows the following key thematic areas relating to adherence counseling quality issues: motivating and educating patients on ART adherence; acting as role models as many of the ASWs are HIV positive; and providing and assuring the availability of specialized adherence counseling services. Both groups emphasized the importance of the counseling patients receive from ASWs.

- *“Reporting of drugs side effects to clinicians by ART clients proved that ASWs are giving correct information to clients” (HCW in FGD).*
- *“ASWs have helped patients to understand the expected outcomes in terms of CD4 counts and other parameters, after commencement of treatment” (ASW in FGD).*

ASWs are likely to form personal bonds with ART patients as a result of their HIV status and treatment status:

- *“As affected/infected people, we offer ‘hands-on’ kind of adherence counseling to clients because we speak from experience and clients feel more encouraged” (ASW in FGD).*
- *“Adherence counseling done by ASWs is more effective as they are also HIV positive and in the same boots as the clients.” (HCW FGD)*
- *“As non-medical people, ASWs are able to remove fears, myths and misconceptions” (HCW in FGD).*
- *“ASWs reinforce adherence counseling on every patient's visit unlike it used to be done before” (HCW in FGD).*

#### Key informant interview results

All the facility managers and ART clinic in-charges interviewed confirmed that ASWs were conducting adherence counseling in their facilities and contributing to the reduction of the workload of HCWs. In addition, the time to prepare a patient before the initiation of ART has reduced as a result of the contribution of ASWs. Three of the five facility managers rated the quality of adherence counseling provided by ASWs to be very good while two said it was good. None said it was unacceptable.

## Discussion

Given the well documented negative effects of non-adherence, there is a growing need in many resource-poor settings to develop practical, cost-effective and feasible adherence support programs [4], [7], [10], [11], [19], [31]. In Zambia, like many of the countries that are rapidly scaling up ART services, this need is particularly dire as a result of the widespread shortages of trained health workers.

The results of the quantitative data showed no significant difference in the proportion of ART clients receiving pre-treatment and follow-up counseling before and after ASWs were introduced. However, the disaggregated results by staff show that there is a significant workload shift from HCWs to ASWs as shown in Tables 3 and 4. Although the number of ART clients increased at each facility over time, the contribution of HCWs and ASWs ensured the majority of patients received pre-treatment counseling, despite the increased patient load. ASWs conducted approximately half of all pre-treatment and follow-up adherence counseling within the facilities. The number of untrained staff (e.g. data entry clerks, registration officers) within the ART clinics who stepped in to provide adherence counseling to support HCWs, declined after ASWs were introduced. The quality of adherence counseling provided by ASWs when compared to HCWs was similar. This was confirmed by the key informant interviews with the facility managers and ART clinic in-charges who rated their performance as either good or very good. These findings were also supported by the comments provided by both the HCWs and the ASWs on the positive role that ASWs are playing in addressing the human resource problem. ASWs were found to be effective in significantly reducing patient waiting times and the workload of HCWs, whose time was then available to perform other important duties at their clinics.

The quality of adherence counseling and positive contribution of ASWs could be attributed to a number of factors. The standardized curriculum with practical attachment coupled with supervision by trained adherence counselors (HCWs) allowed the ASWs to provide good quality adherence counseling service. The fact that most of the ASWs are HIV positive and on ART enables them to discuss drug information, side effects, adherence and other social issues from a more practical and personal standpoint.

A key finding of the FGDs and key informant interviews was the emphasis that respondents placed on the important social support provided by ASWs, not only in helping them take medication, but also helping them handle the emotional and psychosocial aspects of HIV infection. ASWs were found to be unparalleled role models for demonstrating the benefits of treatment adherence. They provide a unique and a personal perspective in dealing with the day-to-day realities of living with HIV that HCWs often cannot provide. In addition, ASWs are often able to forge close relationships with clients, often more candid and comfortable, than the relationships forged by HCWs.

The relatively low training cost and the important contribution that ASWs offer in complementing the efforts of HCWs make it a viable option in addressing human resource constraints in ART programs. The average cost of training one ASW is approximately $320. This cost ranges from $266 to $380 and includes meals, accommodation and transportation. Once trained and assigned to a health facility, each ASW receives a monthly stipend of $25 and volunteers 20 hours per week. ASWs are quite stable with a relatively low attrition rate. Out of the 248 trained by the time of the study, only 22 were not at post due to ill heath or loss to follow-up after 18 months. This pool of trained lay providers with low attrition has the potential of supporting adherence counseling as ART patient numbers increase as highlighted by this study.

In this study several proxy measures were used to assess the quality of adherence counseling including duration of counseling, allowing and responding to questions from clients, ease of understanding the adherence counseling sessions, recall of advantages of ART, drug specific information and safer sex methods. Though some adherence counseling tasks shifted to ASWs, there was no statistical difference in the quality measures before and after the introduction of the ASW program as well as between ASWs and HCWs. In addition, self-reported adherence outcomes between ASWs and HCWs after the introduction of ASWs were similar. These findings do not support perception that lay providers provide inferior quality of service.

Interviewing ART patients within a clinic setting is likely to introduce a selection bias as clients who are interviewed are more likely to be retained in care and treatment. However, analysis of the ARV dispensing tool, which tracks all patients on ART, shows an 85.0% retention rate among all new clients before the introduction of ASWs compared to 100.0% 12 months later. Rosen et al reported that ART programs in Africa are on average retaining 80% of their patients after six months on ART and roughly between 25%–75% after two years depending on the estimating method used [32]. The results from the analysis of the data from the ARV dispensing tool underline a major strength of ASWs, which is their ability to conduct follow-up visits into the community. Patients who have missed clinic or pharmacy appointments are followed-up and counseled in the community by the ASWs. This important finding is corroborated during the FGDs among HCWs with the observation that more ART patients who had adherence problems were followed to their homes and provided with post ART adherence counseling. ASWs provide an additional value at community level since they complement the work of HCWs at facilities and contribute to the reduction of loss to follow-up in the communities.

Although informative, our study has limitations. Out of the 49 ZPCT ART sites, only five were selected because they had backed up electronic information for over 12 months before the introduction of ASWs. Secondly, the adherence measurement was based on patient self-reports which may be affected by desirability and recall bias. Thirdly, this study only assessed the performance of ASWs when working under the supervision of HCWs. However we have drawn on both quantitative and qualitative methods and the findings of the two components to support the conclusion that the quality of adherence counseling does not differ by personnel. Further research is however required as lay providers are mainstreamed into service provision in health facilities.

The study found that ASWs were effective in providing quality adherence counseling, improving patient retention and reducing the loss to follow-up rates as well as addressing the human resource problem at selected hospitals in Zambia.

The findings indicate that the shifting of tasks from more skilled HCWs to ASWs does not compromise the quality of counseling provided. In a resource-limited setting where staff shortages are common, ASWs provide the much needed support for an overburdened workforce. In addition, ASWs who are mostly PLWHA may be in a better position to provide empathic and emotional support to patients as well providing community follow-up which remains a challenge among HCWs. Training of lay providers like ASWs is necessary to help address issues of inadequate human resource associated with ART scale up.
